# Components of Equity-Oriented Health Care System: Perspective of Iranian Nurses

**DOI:** 10.5539/gjhs.v7n2p94

**Published:** 2014-09-28

**Authors:** Zahra Rooddehghan, Alireza Nikbakht Nasrabadi, Zohreh Parsa Yekta

**Affiliations:** 1 School of Nursing and Midwifery, Tehran University of Medical Science, Iran, Tehran

**Keywords:** care, equity, nursing, qualitative research, Iran

## Abstract

Equity in health is one of key objectives in health care systems world wide. This study aimed to explain the perspective of Iranian nurses about equity in the health care system. A qualitative exploratory design with thematic analysis approach was used to collect and analyze data. Using a purposeful sampling helped the researchers to recruit 16 eligible participants. Data were collected via in-depth semi-structured interviews. Five main categories were extracted through data analysis process including (1) inequity against the nurse, (2) the recommended patient, (3) no claim for equity-oriented care in health system, (4) physicians’ dominancy system; and (5) the need to define criteria to measure equity-oriented care. All health care systems around the world struggle to establish equity-oriented care. In perspective of Iranian nurses, the reform of structures in the health system is possible through providing the context of equitable care for caregivers and care recipients. Health system should commit the flow of equity at all of its levels. It should utilize policies to claim equity and consider the interests of all beneficiaries. Furthermore, certain criteria should be defined for equity-oriented care in the health care system, and also provides the possibility to measure and monitor it.

## 1. Introduction

Equity is the most frequent word in social literature of all nations. No social thinker and no government throughout the history have disregarded equity such that despite all conflicts, governments have claimed to support equity. The nature of equity has always been one of the most important questions in human mind and has received highly divergent responses. Practically, human has no brilliant record in this regard (Bagheri Lankarani, Lotfi, & Karimian, 2010).

Equity in health means to achieve the highest possible equity in health for all community. This important indicator is a combination of five sub-categories including community health status, distribution of health in community, health system accountability, health system accountability distribution and equity of financial contribution of households that they can also be a combination of different indicators (Specialized Committee on Health and Biological Sciences, 2010).

Strive for equity in health is one of the clear and key objectives in health systems worldwide. Although most health systems’ access to health services is inequitable, and more services and with better quality are dedicated to rich people and poor people are deprived of the services needed, attempts to establish equity top priority programs of health systems of countries and the World Health Organization (Rechel, Blackburn, Spencer, & Rechel, 2011).

Experience of inequity will mainly lead to resistance, conflict, shock, damage, anxiety, depression and disappointment in community, and in this case people strongly feel that they are betrayed. In fact, inequity itself is considered as a stressor and harmful factor for health and well being. Suffering from inequity may undermine confidence of the public to health system and finally people may stand against it. So, in all cultures, equity like care is known as a need for comprehensive development, health maintenance and human survival (Johnstone, 2011).

Also, in nursing profession equity in service delivery is regarded as one of the values that should always be considered. Equity is a principle in ethics of health-oriented care and is an indicator that infers right, equitable and fair service provision, and equality in being taken care of (Rich & Butts, 2005).

Equity in health care is defined in three situations: (a) equality in access to care, for equal needs, (b) equal use for equal need, and finally, (c) equal quality of care for all people in need (Whitehead, 2000). So, being under equity-oriented care may not guarantee one’s best interest, but equity in health care often provides distributional equity, i.e. the equitable distribution of scarce health care resources (Rich & Butts, 2005).

As in providing care, nurses are frequently exposed to decisions in which a sense of equity or inequity prevails. For example, a nurse who is alone in a ward and a new patient is admitted and another patient needs to take medication for his pain, nurse should weigh up the situation and act on the basis of equity (Blais, Hayey, Kozier, & Erb, 2004). The principle of equity requires that the nurse in equal opportunities act equally and in unequal situations act differently. In other words, patients with similar diagnosis or similar care needs should receive one kind of care and those who have more or less needs should receive a different care type (Chitty & Black, 2007).

In fact, access to health and treatment services is a fundamental right of all human beings (Tofighi, 2010), and factors that endanger human health should be reduced for all people and patients should be able to benefit from the services appropriate to the type and severity of the disease (Bagheri Lankarani et al., 2010). One of the trends expected in 2020 horizon for Iran in nursing group is equitable access for all people in community to safe, efficient, effective, reliable, and high quality nursing services in all health level (Policy Council of Ministry of Health and Medical Education, 2009). However, we observe that care systems have failed to take appropriate measures to establish equity in health system (Bagheri Lankarani et al., 2010). In this study, Iranian participant nurses have raised their views on equity in care system.

## 2. Methods

### 2.1 Aim and Design

A qualitative design based on the thematic analysis approach was used for the data collection and analysis. The aim of this study was to explain the perspective of Iranian nurses about equity in the health care system.

### 2.2 Participant

In this study, 16 nurses (12 females and 4 males) in Iran were selected through purposive sampling, among whom 10 nurses were employed in public hospitals, 4 nurses had experience of working in both public and private hospitals and 2 of them were working in Iranian nursing organization. Sampling was carried out in three cities of Iran. Among participants, seven nurses had been nursing managers in addition to having clinical experiences. In this sam-pling method, the basis of selecting participants was having special information about the considered phenomenon. Sampling continued until data saturation was reached. Therefore, sampling stopped when the data obtained in interviews were repetitive and the answer to the study question was achieved. In this study, sex, work experience and work place were among the important points in achieving maximum variation.

### 2.3 Data Collection

In-depth, semi-structured interviews were used individually and face to face. The average interview time was forty minutes (minimum 30 minutes and maximum 65 minutes). The interviews were held at work or at home according to participant’s request. The interviews were conducted by the first author. Interviews were audio-recorded and transcribed verbatim after each session. After the warm-up phase, the interview was directly followed up with the subject of the study. The major interview questions were the following: what the requirements of an equity-oriented care were? What they do to consider it equity-oriented care? What they do that they consider it not equity-oriented care? What experience they have in this regard? What moves them toward equity-oriented care or moves them away from it? In addition, during interviews, probing questions were used in order to clarify participants’ responses.

### 2.4 Ethical Consideration

Approval for the study was obtained through the ethics committee and the research council affiliated with Tehran University of Medical Sciences (91D1302870). All participants were given information about the study, including the aims and contact details of the researchers. In addition, they were informed that they could withdraw at any time that they wanted without any penalty. Prior to research, written consent forms were taken from nurses. Moreover, their permission was obtained for the recording of the interviews. Also, they were assured that the records will be deleted. Confidentiality was maintained through all steps of data collection and analysis through the allocation of a code for each participant.

### 2.5 Data Analysis

Thematic analysis approach was used to analyze the data. In fact, thematic analysis is a useful and flexible method for qualitative research that searches for themes or patterns (Braun & Clarke, 2006). This method is an analytic approach that leads to organizing and analyzing the data through examining its rich details (Smith & Sparkes, 2009). In this study, the procedure proposed by Braun and Clarke (2006) in thematic analysis was used. In this way, transcribed data were read several times, and initial ideas were noted in order to become familiarized with the data. Then, interesting features of data were coded. After that, two researchers reviewed the codes and put them in potential themes. At the next stage, researchers identified themes and subthemes, made comparisons between the data, and created the thematic map. Finally, an ongoing analysis was conducted to refine the specifics of each theme and to find the overall story of the analysis (Braun & Clarke, 2006).

### 2.6 Rigor

In the data analysis; the involvement of two other researchers in addition to primary researcher enhanced the reliability of the interpretation of the data. These researchers read the transcripts to confirm the coding and categories and checked the primary researcher’s interpretations. Credibility was enhanced through participants’ revision as member checking. Some of the participants were contacted after the analysis and were given a full transcript of their coded interviews to determine whether they were true according to their experiences. The overall level of agreement about peer checking and member checking was above 95%. Maximum variation of sampling and prolonged engagement increased the credibility of data.

## 3. Results

The nurses’ mean age was 44 years. Data analysis resulted in the identification of five themes: (1) inequity against the nurse, (2) the recommended patient, (3) no claim for equity-oriented care in health system, (4) physicians’ dominancy system; and (5) the need to define criteria to measure equity-oriented care. Each category is elaborated separately in the following:

### 3.1 Theme 1. Inequity AboutNurses

In this category, nurses described the current situation and stated that practically there is a far distance between them as caregivers and equity-oriented care system in the care process. They emphasized on nurses’ high working pressure and nurses’ lack of motivation. In response to questions related to equity in care, nurses mentioned their current situation in health system and stated that many obstacles to equity-oriented care resulted from inequity in their own right. Nurses believed that when the nurse-to-bed ratio is not standard, and they have to undergo high working pressure in each working shift, they are forced to provide minimum care to patients, which they call inequity in patient care; for example one of the participants said: *“When I’m responsible for one or two patients, what is considered as the standard, I’d dedicate all my time to them and naturally I can observe equity very much. But when I’m responsible for 8 patients, I can’t do the usual care, let alone think of the equity. At times we have to spend more time for a patient, and the medicine of another patient that should have been taken at 2 pm, would be given to him at 6 pm. I mean you can’t really observe equity. The reason I guess is shortage of nurses”*.

Another nurse working in chemotherapy ward said: *“Nursing service department has ordered us we have to provide care for 8 patients daily, but today 25-26 patients are waiting. How much energy do I have?”*

Also nurses have emphasized the issue that the current conditions for nursing profession is such that nurses experience job burnout and lack of motivation, which will affect providing equity-oriented care to patients. As nurses state, the lack of motivation creates conditions for nurses that inadvertently will lead to inequity in care. In fact, nurses consider the lack of motivation one of the barriers to equity-oriented care. For example one of the nurses said: *“Conditions affect nurses’ spirit in long term, they may affect her capability emotionally and intellectually, and her mood.”*Another nurse said that; *“Nursing motivation has decreased … I think nursing has been underestimated, I mean what nurses do has been disregarded, that’s why its value has gradually decreased and made nurses lose their motivation”*.

### 3.2 Theme 2. The Recommended Patients

Regarding this category, participants stated that achieving an equity-oriented care system needs a structure in which conditions of patient’s admission and care is clear and influential elements cannot put pressure on existing structures through their power. Nurses define a “recommended patient” as a patient who is prioritized without considering the rules and only because of being introduced by an authority, manager, or even an acquaintance receives services and sometimes may get better quality service. Nurses emphasized reform of hospital structures related to admission of patients in this way. Also, nurses stated that the recommended patient would mean a doubling of their work as the person who recommends asks for more care provided for that particular patient. Furthermore, these patients themselves have high expectations of receiving care from nurses. As nurses say such expectations increase working pressure and lead to stress and anxiety for nurses. Therefore, nurses believe recommended patients in health system will result in violation of other patients’ rights and this is in contrast with equity. One of the nurses said: *“When a nurse is doing her job, she likes to treat all patients equally, to see all patients the same, but when a patient has been recommended by an authority, be it your colleague, someone in charge, someone familiar, someone responsible, or anyone recommending him, unknowingly your work for that patient will be different. You should take care of him more carefully, visit him more, pay more attention to his demands, and try to make him and his companions feel more comfy during treatment period”*.

Another participant said: *“The structure itself discriminates between patients. Why such a thing should happen at all? What’s the difference?”*

One of the nurses states about the effect of the recommended patient on nurses: *“For example, when there is a patient with ankle sprain that the chief or manager of the hospital has recommended him, he has additional expectations from nurses who provide care services for him as he has been recommended… he won’t accept our accountability and just says he was that important guy’s relative, why his work was done like that? Why it took so much time? Why it was done so badly? Why a more experienced nurse didn’t come to him?”*

### 3.3 Theme 3. No Claim for Equity-Oriented Care in the Health System

Nurses stated that health system does not claim equity-oriented care from them and this matter is disregarded, therefore organization has no contribution in moving nurses toward equity-oriented care. Nurses pointed that sometimes the organization itself moves nurses toward inequity. Also, nurses mention cases where following equity has resulted in their loss. So, to access equity-oriented care system, the policies of health system should change toward claiming equity.

One of the nurses said: *“I think in our four years of education, this issue that nurses should treat all patients equally is just dealt with verbally, though we swear we’d treat all patients equally and when we enter the hospital environment we’re just told that all patients, poor and rich, are the same. But in practice it’s not so and even the authorities themselves want these differences to be made”*.

One of the nurses said: *“If a nurse wants to observe the so called equity among patients, she may get hurt financially or socially”*.

### 3.4 Theme 4. Physicians’ Dominancy System

Participants mentioned that when the health system is in the hand of a particular group of people and it is run by them and their interests are always prioritized over those of others, equity in the health system and consequently in the care system will not be realized. The reason is that there is no place for other beneficiaries in this management system, so how such a health system can claim equity in the health system?

On this issue one of the nurses said: *“this is not fair that in our country all managerial positions are for our doctor colleagues”*. Another participant said: *“The biggest problem in nursing system is that our authorities are doctors, in all ranks. Now I’m here as a head nurse, nursing office is directly responsible for me, hospital matron is directly responsible for me, I’m selected and offered by nursing office, this office knows my job, and supervises my work, but my assignment letter should be signed by the hospital president, this is the main physicians’ dominancy system”*.

### 3.5 Theme 5. The Need to Define Criteria to Measure Equity-Oriented Care

The necessity to define measures of equity-oriented care is the title of the fifth category. In this study, it was determined that each of the participants consider equity-oriented care based on personal opinions and views and their beliefs and this means there is no clear definition of equity-oriented care is the health system. If the definition of equitable care is ambiguous, there will not be an opportunity to review and assess equity in care. Therefore, achieving an equity-oriented care system requires a clear definition of equity-oriented care in the health system.

Nurses have defined it based on various dimensions and these definitions consider care, care recipient, caregiver, the comparison between care recipients and both care spectra i.e. the nurse and patient. The difference of views can be seen in the following statements: *“If services are appropriate and timely, the nurse can claim she’s observed equity”; “If the patient’s rights are observed, and the relationship between nurse and patient is fine, I think it will result in more equity”; “I think equity in care is performed when a nurse has a clear conscience and a patient is fully satisfied in the ward; I mean if the nurse is conscientious and is satisfied with her work and the way of her caring, and if the patient is satisfied”; & “It’s patient’s luck he is admitted to which ward or shift where its personnel are not knowledgeable, skilled or conscientious as compared to another patient with the same disease and conditions! The patient only says: this is my chance; I should be here when it’s the time of this personnel shift!”*

## 4. Discussion

In this study, Iranian nurses have considered the need to reform the structural inequity related to nurses. In their view, the care system will be equitable when nurses as caregivers will not be oppressed and equity will be observed for them so that they will be able to provide equity-oriented care for patients. In fact, nurses believe that inequitable conditions for the nurse will lead to inequity for the patient and as long as nurses themselves have not felt the equity, they will not be able to consider equity in providing care for patients. Examples of this structural inequity in their view include high work pressure, not observing the standard nurse-to-patient ratio, the lack of using nurses based on their ability and skills etc that highly affect the care provided by them.

In a study that Golparvar and Nadi(2010) conducted on 478 male and female nurses employed in hospitals of Isfahan University of Medical Sciences, titled “the relationship between understanding equity and nurses’ customer-oriented behaviors”, they found that there is a positive and significant correlation between perceived distributional equity and perceived procedural equity and customer-oriented behavior among nurses. This means that nurses’ actions for clients are affected primarily by hospital’s equity in payments, rewards and facilities, and a fair schedule for them. When they feel that procedures of decision-making are fair for them and their conditions are equitable, their cognition of observing equity in allocating the outcomes will increase, hence they treat patients better.

However, in many parts of the world, nurses earn less than their counterpart physicians despite passing higher education and gaining further qualifications. They are oppressed in that they earn unfairly inadequately (Lee & Saeed, 2001; Hagbaghery, Salsali, & Ahmadi, 2004). Therefore, their decreased organizational commitment all over the world has led to many organizational problems such as the increased turnover, frequent absenteeism, low job motivation, reduced performance of nurses and consequently lowers performance of hospitals that eventually will lead to the loss of care services and efficiency of hospitals (PPE, 2007).

In a study conducted Bakhshi, Kumar, and Rani (2009) perceived organizational equity was examined as a predictor of job satisfaction and organizational commitment. In this study, distributional equity, procedural equity and job satisfaction were measured in 128 employees of medical academic staff from India. Regression analysis of the data showed that distributional equity has a significant relationship with job satisfaction, while procedural equity has no significant relationship with job satisfaction. Both distributional and procedural equity have a significant relationship with organizational commitment.

In a literature review conducted by Dinmohammadi, Hushmand, Cheraghi, and Peyrovi (2012) on 31 papers about oppression in nursing, it was found that oppression is a phenomenon experienced and reported by most nurses all over the world. This phenomenon is formed by determining a set of norms from the dominant group and a belief that the people outside this group are subordinate. The main features of oppression include inequitable treatment, ignoring the rights of people and degrading human dignity; and hierarchical structures in hospitals provide favorable conditions for this phenomenon and will lead to damaging effects among nurses.

Iranian nurses experienced difficulties relating to work settings and difficulties relating to socio cultural factors in nursing. Difficulties related to work settings included personnel shortages, heavy workloads, unclear tasks, lack of registered and auxiliary nurses, equipment deficiencies and low salary. Dissatisfaction with the content of their work had also contributed to feelings of hopelessness and frustration among many Iranian nurses. Difficulties relating to socio cultural factors included poor public image and a low social status in Iran (Nikbakht Nasrabadi & Emami, 2006; Nikbakht Nasrabadi, Emami, & Parsa-Yekta, 2003)

Another argument by nurses is about the recommended patient. That is, some people have power and influence in the health system, so they do not consider existing structures, and provide the available facilities for patients who are their friends, while these patients have no medical and logical preference over other patients who do not have an influential person in the health system. Chitty and Black (2007) wrote the principle of equity implies that in equal conditions should act equally and in unequal conditions should act unequally. So, prioritizing patient and taking more care of him because of having a connection in the health system is not compatible with the principle of equity and is in contrast with the equity-oriented care system.

Another theme emerged from nurses’ statements is no claim for equity-oriented care in the health system. Marquis and Huston (2012) write all organizations need to establish facilities for extensive policies and procedures to direct staff practices. Unfortunately, in many health care organizations, this function falls to isolated policy and procedures committee. While it is expected that each organization makes clear how to achieve goals according to its philosophy and general and objective goals in the field of the organization’s activities. These policies direct people’s behavior toward the organization’s mission.

Therefore, to achieve an equity-oriented care system it is necessary to formulate policies consistent with this goal to move caregivers to an equity-oriented care. An equity-oriented care system will not be achieved without reforming health systems policies to achieve equity.

Furthermore, nurses noted that health system should consider benefits of all beneficiaries from the top to bottom of health system and not just the interests of a particular group i.e. doctors are considered. As long as policies are such that only interests of a certain group are included, it cannot be expected to achieve an equity-oriented care system, as physicians’ dominancy system in the health system will highly affect the practice of other groups in the health system such as caregivers.

Studies have also shown that paternalistic organizational structure in most care systems affects nurses’ ability to perform their duties. These conditions result in more oppressive conditions day by day, and have been addressed from different angles (Dong & Temple, 2011; Fletcher, 2006).

Another issue addressed in nurses’ statements is the necessity to define criteria to measures of equity-oriented care. In fact, the lack of a specific scale for measuring equity-oriented care will lead to the fact that each nurse would consider equity in care based on her beliefs, and definition of equity. This means ambiguity in definition and measurement of equity-oriented care.

On this issue Whitehead (2000) writes: The concept of equity in relation to health and health care can have a variety of meanings for different people. However, formulating practical policies to reduce inequity should begin when the concept of equity in health is well defined (Farshad, 2009). Bagheri Lankarani, Lotfi, and Karimian (2010) believe many health systems have not mainly attempted to monitor inequities in their care system, and are not aware of the existence of such gaps in their care system, so it is necessary to accurately assess these criteria in the health system.

In view of Iranian nurses, requirements of realization of equity-oriented care system for all health systems is provided through the preparationbackground of equity-oriented care for caregivers and care recipients, claim for equity by the health system, considering the interests of all beneficiaries and finally determining criteria to measures of equity-oriented care in health system.

## 5. Conclusion

In fact, all health systems should plan practical steps to achieve equity in care system. To accomplish this, the health system should commit the flow of equity at all of its levels through the establishment of an equitable structure for caregivers and care recipients. It should utilize policies to claim equity and consider the interests of all beneficiaries. Furthermore, certain criteria should be defined for equity-oriented care in the care system that not only care staff is committed to regardless of their personal beliefs, but also it provides the possibility to measure and monitor an equity-oriented care. In equity-oriented health system, nurses are more satisfied, and also provide more qualified care.
